# Ligand based lead generation - considering chemical accessibility in rescaffolding approaches via BROOD

**DOI:** 10.1186/1758-2946-4-S1-O20

**Published:** 2012-05-01

**Authors:** Li-hsing Wang, Andreas Evers, Peter Monecke, Thorsten Naumann

**Affiliations:** 1 Sanofi-Aventis Deutschland GmbH, Industriepark Höchst / G838, D-65926 Frankfurt, Germany

## 

In pharmaceutical industry ligand based approaches like scaffold hopping, scaffold decoration and me-too approaches, are used to generate lead structures in discovery projects. We use several tools to generate novel lead structures, such as BROOD [1]. BROOD is a software tool which explores chemical space around query molecules based on shape similarity and electrostatics, and it generates analogs of a reference molecule by replacing a selected moiety with fragments from *in silico* fragment databases. The content of these fragment databases has an essential influence on the molecules generated by BROOD. Due to the amount of resources required to synthesize novel chemical compounds, an easy access to chemical compounds is crucial for the broad applicability and for the acceptance of *in silico* approaches which propose novel molecules to the synthetic chemist. In order to consider synthetic accessibility of *in silico* generated molecules, we use our inhouse libraries of drug-like and chemically feasible molecules for the *in silico* fragment generation. In addition, we implemented fragmentation rules which reflect the (retro-)synthetic access to these molecules. The combination of (retro-)synthesis rules and fragments of existing compounds leads mainly to synthetically accessible compound proposals. To identify relevant available compounds, we implemented an approach where *in silico* generated molecules are used as search queries to search in inhouse available compound libraries, e.g. via ROCS [2]. This approach leads directly to internally existing compounds which can be ordered for experimental testing. However, if novel chemical matter is desired, chemical synthesis is necessary. Therefore, as a further extension of our approach, we started from available chemical reagents as input for our *in silico* fragment databases. The available reagents are detected by well-defined chemical reactions, converted to fragments and stored in the *in silico* fragment databases. As the chemical reactions are considered during the virtual synthesis step, the synthetic accessibility of compound proposals is increased. We present the concept of these approaches, examples and typical applications for different targets.
